# At the interface of antioxidant signalling and cellular function: Key polyphenol effects

**DOI:** 10.1002/mnfr.201500940

**Published:** 2016-03-29

**Authors:** Asimina Kerimi, Gary Williamson

**Affiliations:** ^1^ School of Food Science and NutritionUniversity of LeedsLeedsUK

**Keywords:** Endothelial function, Flavonoid, Inflammation, Phenolic acid, Uric acid

## Abstract

The hypothesis that dietary (poly)phenols promote well‐being by improving chronic disease‐risk biomarkers, such as endothelial dysfunction, chronic inflammation and plasma uric acid, is the subject of intense current research, involving human interventions studies, animal models and in vitro mechanistic work. The original claim that benefits were due to the direct antioxidant properties of (poly)phenols has been mostly superseded by detailed mechanistic studies on specific molecular targets. Nevertheless, many proposed mechanisms in vivo and in vitro are due to modulation of oxidative processes, often involving binding to specific proteins and effects on cell signalling. We review the molecular mechanisms for 3 actions of (poly)phenols on oxidative processes where there is evidence in vivo from human intervention or animal studies. (1) Effects of (poly) phenols on pathways of chronic inflammation leading to prevention of some of the damaging effects associated with the metabolic syndrome. (2) Interaction of (poly)phenols with endothelial cells and smooth muscle cells, leading to effects on blood pressure and endothelial dysfunction, and consequent reduction in cardiovascular disease risk. (3) The inhibition of xanthine oxidoreductase leading to modulation of intracellular superoxide and plasma uric acid, a risk factor for developing type 2 diabetes.

**Abbreviations**
AP‐1: activation protein
AT: adipose tissue
ECM: extracellular matrix
EDH: endothelium‐dependent hyperpolarization
EGCG: epigallocatechin gallate
eNOS: endothelial nitric oxide synthase
ERK: extracellular signal‐regulated kinase
ET‐1: endothelin‐1
GDH: glutamate dehydrogenase
HUVEC: human umbilical vein endothelial cell
IRs: insulin receptors
IRS‐1: insulin receptor substate‐1
JNK: c‐jun N‐terminal kinase
MAPK: mitogen‐activated protein kinases
MMP: matrix metalloproteinase
NEFA: non‐esterified fatty acids
NF‐κB: nuclear factor κB
NOS: NO synthase
NOX: NADPH oxidase
Nrf2: NF‐E2–related factor 2
PI3K: phosphoinositide‐3‐kinases
ROS: reactive oxygen species
sE‐selectin: soluble endothelial selectin
SIRT: sirtuin
TLR: Toll‐like recognition receptor
TNF: tumour necrosis factor
VEGF: vascular endothelial growth factor
VSMC: vascular smooth muscle cells
XOR: xanthine oxidoreductase

## 1 Introduction

The increasing occurrence of nutrition‐related diseases collectively clustered under the term metabolic syndrome, and the spiralling cost of clinical treatment, has resulted in heightened interest in plant bioactives and their postulated protective role as part of a healthy diet. For many years, the chemical action of polyphenols, flavonoids, “tannins” and phenolic acids (referred to here collectively as (poly)phenols) as antioxidants was considered to be the primary mechanism of action in vivo to explain epidemiological and intervention studies, animal studies, and popular interpretations such as the “French paradox” or the “Mediterranean diet” 1. Chemical antioxidant activity can be readily measured in vitro, but, in the last few years, it has become clear that the effects of (poly)phenols in vivo are due to much more complex modes of action 2, with multiple but modest effects on different targets, adding up to a substantial overall response. These pleiotropic effects reported in the literature point to numerous potential targets of (poly)phenols derived mainly from cell studies and animal models, but only some of these are backed up by human intervention studies. Although the direct action of (poly)phenols in vivo as purely chemical antioxidants is mostly discredited, many of the proposed mechanisms in vivo are related to oxidative processes, involved in cell signalling, inflammation, insulin resistance, glucose and lipid metabolism, mitochondrial function and superoxide production. Data from recent studies suggests that the efficiency of dietary bioactives in modulating plasma biomarkers also depends on the health status of individuals. In fact, in some cases the most positive results are obtained in people already with some increased risk factors for cardiometabolic disease. Healthy subjects with low levels of oxidative stress showed a reduced responsiveness to nutritional interventions 3. Here, we consider how the “antioxidant hypothesis” is now maturing, allowing relevant mechanisms of action to be discovered in vitro and then confirmed in vivo, and also for efficacy data from in vivo intervention studies to be explained mechanistically from in vitro data. To address these issues, we have taken several examples where human intervention data have provided evidence of real effects, and where there is mechanistic data from in vitro or animal models to support the data.

After oral consumption, there are several possible pathways of absorption and metabolism of (poly)phenols depending on the chemical structure and nature of attached moieties. These have been reviewed extensively elsewhere and are quite well understood 4, 5. After consumption of phenolic acids such as chlorogenic acid in coffee, the resulting predominant compounds in the blood are ferulic acid‐*O*‐sulfate, dihydroferulic acid‐*O*‐sulfate, dihydroferulic acid and dimethoxycinnamic acid, along with numerous other less abundant compounds 6, 7. Following consumption of quercetin or (–) epicatechin, *O*‐sulfated, *O*‐glucuronidated and *O*‐methylated forms appear in plasma fairly rapidly, followed by various microbiota‐derived phenolic compounds and their conjugates at later times 8, 9. It should be noted that flavanols contain stereochemical centres giving rise to different enantiomeric forms, denoted as (+) and (–). The naturally occurring form of epicatechin is (–)‐epicatechin (and derivatives) and of catechin is (+)‐catechin, and these are indicated when it is clear which form was used. However, some processing and extraction techniques can cause formation of (+)‐epicatechin and of (–)‐catechin 10, which are not resolved on a reverse phase HPLC column, and therefore not readily observed 11. Therefore some of the effects ascribed to (epi)catechins in this report may be due to enantiomeric mixtures, if not explicitly stated. This may be important since the enantiomers may have different bioavailabilities 12 and bioactivities 13. Microbial metabolites, after consumption of berries rich in multiple (poly)phenols, reached high concentrations up to 20 μM in the blood 14. Anthocyanins and procyanidins are poorly absorbed in their intact form, but microbial and chemical breakdown products are well absorbed and appear in plasma and urine 15, 16. The extensively studied (poly)phenol resveratrol, on the other hand, is quickly absorbed but rapidly metabolised resulting in low bioavailability of the parent compound 17. The complexity and diversity of compound structures poses a challenge when designing in vitro experiments, where cells are treated with relevant concentrations and chemical forms of (poly)phenols and metabolites compared to normal dietary consumption of (poly)phenols. Previously, we have recommended strategies for tackling this issue 18. Treating with single compounds in vitro does not take into account the complex mixture of plasma metabolites that could have additive, antagonistic or even synergistic effects. An alternative approach is to first undertake intervention studies and determine an “effect,” i.e. a change in a relevant biomarker. Ideally the study should be conducted by using high (poly)phenol‐containing food, supplements or pure (poly)phenols on a well‐defined cohort, preferably monitoring some sub‐optimal biomarkers. If an improvement in the chosen biomarker is observed in vivo, then the mechanism should be further studied in vitro. This approach helps to circumvent the arguments concerning which concentrations or forms to use in vitro, since the goal is not to extrapolate an in vivo effect directly from the in vitro studies. Nevertheless, the most relevant effects will be those occurring at the lowest concentrations of the bioactive (conjugate/metabolites), taking into account the pleiotropic effects to allow for multiple mechanisms of action. We have examined this alternative approach here for three examples, chronic inflammation, endothelial effects and plasma uric acid, where (poly)phenols have been shown to have an effect in vivo, and we consider the in vitro data as a way of substantiating the mechanism. This “nutritional” approach is different to that followed in drug design, where the first step is in vitro screening to identify active compounds, which are then ultimately taken to phase II and III trials in vivo. It should be noted that nutrition is fundamentally different to drug discovery in that we are already consuming (poly)phenols through the diet everyday as part of normal nutrition especially with a healthy lifestyle. This approach also takes into account interactions with nutrients and with the host, such as attenuating or blunting post‐prandial sugar spikes or targets of microbial metabolites, which in a previous review we argue are very important when assessing the action of (poly)phenols in vivo 19.

## 2 Effects of (poly)phenols on chronic inflammation

Inflammation followed by infection or tissue injury constitutes a physiological response to harmful stimuli and is meant to aid towards re‐establishment of homeostasis. The classical acute inflammatory response involves the delivery of plasma components and leucocytes to the site of insult and leads to the production of different types of inflammatory mediators grouped as chemokines, cytokines, vasoactive amines, eicosanoids and products of proteolytic cascades that can act as endothelial constriction agents 20. However, the extent of the inflammatory activation characterising the preceding state of diabetes or subsequently the development of the metabolic syndrome is not as large, and hence is termed “low grade” or “low‐intensity” chronic inflammation, evidenced by an increased concentration of circulating inflammatory mediators in an asymptomatic form 21.

Very few human studies have been reported where the effects of (poly)phenols on inflammation are the primary outcome. Several studies have reported effects on inflammatory markers, but only under certain conditions. In a randomized, double‐blind, placebo‐controlled crossover trial involving healthy (pre)hypertensive volunteers, (–)‐epicatechin (100 mg/d for 4 weeks) changed soluble endothelial selectin (sE‐selectin) but the change in z score for endothelial dysfunction was only a trend. In the same study, quercetin‐3‐*O*‐glucoside (160 mg/d for 4 weeks) changed sE‐selectin and IL‐1β, and reduced the z score for inflammation 22. Quercetin aglycone (500 mg/day) given to women for 10 weeks decreased systolic, but not diastolic, blood pressure, but had no effect on IL‐6, α‐TNF and C‐reactive protein compared to the placebo 23. Acute ingestion of 1000 mg quercetin, 120 mg epigallocatechin 3‐gallate, 400 mg quercetin‐3‐*O*‐glucoside, 400 mg eicosapentaenoic acid, 400 mg docosahexaenoic acid, 1000 mg vitamin C and 40 mg niacinamide, 15 min before heavy exercise in endurance athletes, did not affect post‐exercise inflammatory markers nor immune changes 24. In community‐dwelling adult females, quercetin, at 500 and 1000 mg/day for 12 weeks, produced no effect on innate immune function or inflammation 25.

In metabolic syndrome volunteers, hesperidin (500 mg/d for 3 weeks) increased FMD, and reduced blood CRP, and sE‐selectin 26. Also in metabolic syndrome subjects, red orange juice containing 160 mg hesperidin for 1 week improved FMD, blood CRP, IL‐6 and α‐TNF, but not plasma NO concentration 27, while in moderately overweight men, hesperidin decreased diastolic blood pressure and improved microvascular endothelial reactivity post‐prandially at the peak of hesperetin plasma concentration 28, and these changes were associated with a more anti‐inflammatory blood leukocyte gene expression profile 29. Human and animal studies on hesperidin have been reviewed 30. In 42 volunteers with high cardiovascular risk factors, cocoa powder (40 g/day for 4 weeks) decreased serum P‐selectin and intercellular adhesion molecule‐1, and also the expression of VLA‐4, CD40 and CD36 in monocytes 31. Also in subjects at high CVD risk, bilberry juice decreased plasma CRP, IL‐6, IL‐15 and INF‐gamma‐induced monokines, but increased α‐TNF, interpreted as effects on nuclear factor κB (NF‐κB) 32.

### 2.1 Inflammation‐related signalling pathways and kinase cascades

Most known signalling pathways related to inflammation involve members of the IL‐1 and tumor necrosis factor (TNF) receptor families and the Toll‐like recognition receptors (TLR) 33. Activation of either of these receptors recruits adaptor proteins that further recruit signalling proteins, leading to activation of mitogen‐activated protein kinases (MAPK) such as extracellular response kinases (ERK 1/2), jun N‐terminal kinase (JNK), p38 MAPK and inhibitor of nuclear factor κB kinases (IKK). MAPKs lead to phosphorylation of transcription factors such as activation protein (AP‐1) and cAMP response element‐binding proteins that bind to promoters of pro‐inflammatory genes activating their transcription. In addition, MAPKs are able to induce post‐transcriptional mechanisms to directly regulate pro‐inflammatory gene expression. IKK is mainly related to NF‐κB activation, recognised as a central player in inflammatory and immune responses 34, 35. Both MAPK and IKK induce expression of IL‐1β and TNFα, further amplifying the immune response within minutes 36 while TLR activation also results in synthesis of other pro‐inflammatory factors such as IL‐6 and IL‐8. Both cytokine receptor‐ and TLR receptor‐activation leads to enhancement of the phosphoinositide‐3‐kinase (PI3K) pathway and other related kinases such as Akt. Activation of Akt elicits several cellular events such as stimulation of glycogen synthesis, inhibition of gluconeogenesis and stimulation of glucose uptake 37 and is involved in the insulin signalling cascade.

(Poly)phenols can influence these processes in several ways. For example, some lines of research suggest that inactivation of the PI3K/Akt pathway by quercetin can take place via direct inhibition of PI3K based on X‐ray crystallographic analysis showing that quercetin fits into the ATP‐binding site of PI3Kγ with a K_d_ value of 0.28 μM. The direct binding of PI3K by quercetin inhibits downstream the PI3K/Akt pathway of signalling, including inhibition of AP‐1 and suppression of NF‐κB activation 38. In addition, MAPKs are involved in vascular inflammation through the modulation of adhesion molecules (ICAM‐1, VCAM‐1, E‐selectin) and chemotactic factor expression. Several studies showed that (poly)phenols such as resveratrol 39 and piceatannol 40 regulate these events through extracellular signal‐regulated kinase (ERK) 1/2 and p38, and ERK 1/2 inhibition, respectively. Structural studies indicate that quercetin can also have an effect on the MAPK/ERK pathway due to binding to a pocket separate from, but adjacent to, the ATP‐binding site of MAP2K1 41. This interaction would lead to the inhibition of phorbol ester‐induced phosphorylation of ERK and downstream suppression of the activation of AP‐1 and NF‐κB. Hesperetin‐3ʹ‐*O*‐sulfate, hesperetin‐3ʹ‐*O*‐glucuronide and naringenin‐4ʹ‐*O*‐glucuronide (2 μM) significantly attenuated monocyte adhesion to TNFα‐activated human umbilical vein endothelial cells (HUVECs) 42. 4ʹ‐*O*‐methyl (–)‐epicatechin, its glucuronide and EC‐4ʹ‐*O*‐sulfate (0.2–1 μM), attenuated the adhesion of monocytes to endothelial cells by inhibition of NF‐κB and MAPK phosphorylation 43. In HUVECs, (+)‐catechin, (–)‐epicatechin, quercetin and resveratrol (0.001–10 μM) up‐regulated both tissue‐type plasminogen activator (tPA) and urokinase‐type PA (u‐PA) mRNA, resulting in sustained increased expression of surface‐localized fibrinolytic activity 44. TNFα induced up‐regulation of VCAM‐1, ICAM‐1 and monocyte chemotactic protein‐1 (MCP‐1) and mRNA in human umbilical artery smooth muscle cells was attenuated by quercetin, but not quercetin‐3′‐*O*‐sulfate, quercetin‐3‐*O*‐glucuronide and 3′‐methylquercetin‐3‐*O*‐glucuronide 45. In cultured rat aortic smooth muscle cells, quercetin‐3‐*O*‐glucuronide (10 μM) inhibited platelet‐derived growth factor‐induced cell migration and kinase activation 46. Quercetin (15 μM) reduced the monocyte‐binding properties of human aortic endothelial cells 47. In HepG2 cells, (–)‐epicatechin (10 μM) up‐regulated NF‐κB‐binding activity 48, quercetin at the same concentration inhibited H_2_O_2_‐induced NF‐κB activity 49, and quercetin attenuated NF‐κB activation induced by TNF 50. Quercetin protected β‐cells from cytokine‐induced damage partly through NF‐κB 51. In animal studies, a high doses of quercetin delivered i.p. attenuated NF‐κB activation in the liver of streptozotocin‐diabetic rats 52, and inhibited colitis in sodium dextran sulfate‐treated rats 53.

### 2.2 Effects involving adipose tissue

Adipose tissue (AT) is considered an active endocrine organ able to send and respond to signals in order to modulate appetite, energy intake, insulin sensitivity, the endocrine and reproductive system, bone metabolism and inflammation, among other functions 21, 54, 55. It has been suggested that reduction in weight and body fat percentage can decrease the concentration of circulating pro‐inflammatory agents and increase anti‐inflammatory molecules. Metabolically, the dense network of blood vessels present in AT serves for the systematic transport of lipids towards the adipocytes. On the other hand, these vessels also carry adipokines and adipocyte nutrients to meet the metabolic and physiological demands, and in fact pro‐inflammatory signalling in the adipocyte is required for proper adipose tissue re‐modelling and expansion 56. However, in obesity, lipid storage capacity is reduced and enlargement of visceral adipose tissue is prominently affected, especially in the postprandial period. This process probably occurs because the volume of adipocytes in obese individuals has already reached its limit and cannot accumulate TAG efficiently 57. In addition there is an increase in the rate of lipolysis during the fasting period 55. Non‐esterifed fatty acids (NEFA) transport in hypertrophic adipocytes is reduced possibly due to the lack of stimulus generated by insulin and linked to less insulin receptors (IRs) exposed on the cell surface and restricted Glut4 translocation to the cell membrane. As a result, other tissues are exposed to excessive NEFA influx 56.

Adipokines excreted by the AT comprise proteins related to the immune system (e.g. TNF‐α and IL‐6), growth factors (e.g. transforming growth factor‐β) and proteins of the alternative complement pathway (adipsin). Others are involved in blood pressure regulation (angiotensinogen), blood clotting (plasminogen activator inhibitor‐1; PAI‐1), glucose homeostasis (adiponectin, resistin, visfatin and leptin) and angiogenesis (vascular endothelial growth factor; VEGF), among several other actions 58. Besides the fact that AT performs its own metabolic activities, the adipocytes release signals to other tissues and regulate their metabolism. Two mechanisms are suggested in the literature to explain the low‐intensity chronic inflammatory process in relation to AT; adipocyte hypoxia and macrophage infiltration into AT 59. It is hypothesised that the onset of hypoxia stimulates the release of inflammatory cytokines, chemokines and angiogenic factors (VEGF) as a way to increase the blood flow and stimulate vascularisation. Increased expression of adhesion molecules in the endothelium of blood vessels, such as MCP‐1, leads to an increased number of circulating monocytes that infiltrate into AT. In high glucose or advanced glycation end products, quercetin, epigallocatechin gallate (EGCG) and curcumin affected membrane fluidity and transmembrane potential of HUVECs and Jurkat T lymphoblasts, and attenuated the release of pro‐inflammatory factors, such as MCP1 60.

On the other hand, macrophage infiltration can be an important source of pro‐inflammatory adipokines synthesised locally 61, thought to be mainly triggered by stress of the endoplasmic reticulum. It has been shown that TNFα inhibits the differentiation of adipocyte precursors and, similarly, IL‐6 also inhibits adipocyte differentiation causing a vicious cycle of increased NEFA in blood. Increasing concentrations of NEFA are implicated in insulin resistance, also through effects on insulin receptor substate‐1 (IRS‐1) decrease involving IRS‐1 phosphorylation (ser307) catalysed by IKK and JNK. In adipocyte cells, PKC*θ* was also found to be activated by fatty acids and contribute to IKK and JNK activation responsible for IRS‐1 serine phosphorylation and degradation 62. The activation of the novel PKC isoforms depends on the increase of diacylglycerol in the intracellular compartment, which is induced by increased lipid uptake. Upon activation, PKCε/ PKCδ/PKCθ can catalyse the serine phosphorylation of IRS‐1 in muscle (PKCδ and PKCθ) and liver (PKCε), leading to the insulin resistance phenotype 63. Although adipose tissue only accounts for about 10% of insulin stimulated glucose disposal, it has a key role in directing whole‐body glucose homeostasis and two plausible mechanisms have been postulated to explain this attribute. According to clinical data, pharmacological activation of PPARγ in adipose tissue improves its ability to store lipids; therefore it may be assumed it reduces the lipid burden and associated reactive oxygen species (ROS) in muscle and liver. This model involves activation of genes encoding molecules that promote a combination of lipid storage and lipogenesis leading to body‐wide lipid repartitioning by increasing the triglyceride content of adipose tissue and lowering free fatty acids and triglycerides in the circulation, liver and muscle, thereby improving insulin sensitivity 64. On another front, PPARγ‐specific drugs alter the release of signalling molecules from fat, including leptin, TNFα, resistin and adiponectin, which by virtue of serum transport have far‐reaching metabolic effects in other tissues 59.

Effects of polyphenols on PPARγ and downstream pathways have accumulated mainly from in vitro and animal studies (reviewed in 65). Quercetin (IC_50_ = 3.0 μM) and luteolin (IC_50_ = 7.2 μM) were PPARγ antagonists at relatively low concentrations 66 based on an in vitro fluorescence competitive‐binding assay, while mixtures of the aforementioned bioactives and others from an oregano extract were found to activate endothelial nitric oxide synthase (eNOS) dose dependently in HUVECs 66. In human primary adipocytes, TNFα induced IL‐6, IL‐1b and IL‐8, for example. Quercetin (10–60 μM) attenuated this through effects on phosphorylation of ERK1/2 and JNK, NF‐κB‐related transcriptional activity, PPARγ and serine phosphorylation of IRS‐1 and protein tyrosine phosphatase‐1B mRNA expression and its suppression of insulin‐stimulated glucose uptake 67. Mochizuki et al. 68 found that the vascular permeability of quercetin‐3‐*O*‐glucuronide in human aortic endothelial cell culture inserts was dramatically increased by stimulation of interleukin (IL‐1)‐α, an inflammatory cytokine originating from macrophages, while in a study on mice, quercetin attenuated mast cell and macrophage infiltration into epididymal adipose tissue following a high fat diet 69. Supplementation with 0.l% quercetin for 12 weeks modified the phenotype ratio of M1/M2 macrophages, lowered the levels of pro‐inflammatory cytokines, and inhibited polarization and inflammation of mouse bone marrow‐derived macrophages through an AMPKα1/SIRT1‐mediated mechanism. According to others, the anti‐inflammatory action of quercetin in the macrophages is due to inhibition of JNK activation during lipopolysaccharide‐induced mitochondrial dysfunction in an adipocyte cell model 70, 71. Overman et al. 72 ascribed these anti‐inflammatory effects to suppression of NF‐κB activation in agreement with other findings 67 reporting a decrease in TNFα‐induced NF‐κB transcriptional activity in primary human adipocytes and further attenuation of the TNFα‐mediated suppression of PPARγ activity and PPARγ target genes. Similar effects were found for EGCG in macrophage foam cells 73 and these were linked to suppressed expression of CD36, a lipid co‐transporter and a postulated receptor for lipoproteins. In rats, green tea polyphenols reduced fat deposits induced by a high fat diet, ameliorated hypo‐adiponectinaemia and relieved high glucose‐induced adiponectin decrease in visceral AT in vitro. The signalling pathway analysis indicated that PPARγ regulation mediated via the ERK1/2 pathway was involved 74. (–)‐Catechin, not naturally present but produced by heat treatment of green tea, was the most potent of the eight green tea (poly)phenols evaluated in promoting adipocyte differentiation in human bone marrow mesenchymal stem cells in a dose‐dependent manner (1–10 μM) through PPARγ transactivation 75. The multifaceted role of PPARγ on the interface of inflammation and obesity makes it difficult to dissect whether effects of bioactives related to this pathway act by enhancing downstream metabolic biomarkers or by improving the clinical signature of inflammation. In one of very few such human studies, the effect of consumption of polyphenol‐rich cloudy apple juice (containing 40 mg total flavanols, 71 mg dihydrochalcone derivatives, 169 mg total phenolic acids and 4 mg total flavonols, daily for 4 weeks) on plasma parameters related to the obesity phenotype and potential effects of interactions between the apple juice and allelic variants in obesity candidate genes (PPARγ related) was assessed. The apple juice had no significant effect on plasma lipids, plasma adipokine and cytokine levels; however, it significantly reduced percent body fat. The IL‐6‐174 G/C polymorphism showed an interaction with body fat reduction induced by the apple juice. Solely in C/C, but not in G/C or G/G variants, a significant reduction in body fat after 4 weeks of apple juice intervention was detectable 76. This study highlights the importance of inter‐individual variability in nutritional interventions.

### 2.3 Oxidative mechanisms, insulin signalling and mitochondrial dysfunction

It is well known that oxidative stress is a common characteristic in people with the metabolic syndrome phenotype 77. The mechanisms involved in elevation of oxidative stress and inflammatory burden include increased production of superoxide anion via the NAD(P)H pathway, deregulation of leptin levels in adipocytes that stimulate directly ROS production such as hydrogen peroxide (H_2_O_2_), hydroxyl radicals (HO˙) and mitochondria‐related metabolism of energy substrates. Furthermore, sustained high glucose levels lead to generation of advanced glycation end products which accumulate in the vessel wall, and, through interactions with its receptors, activate NF‐κB via MAPKs and lead to the transcription of pro‐inflammatory factors (reviewed in 78). Excess energy substrate in the form of glucose or fatty acids enter the citric acid cycle resulting in the generation of excess mitochondrial NADH and consequently ROS 79. When excessive NADH cannot be dissipated by oxidative phosphorylation, the mitochondrial proton gradient increases and single electrons are transferred to molecular oxygen forming superoxide anions. Although low levels of H_2_O_2_ may be required for normal cellular functioning, for instance to terminate PI3K signalling through protein tyrosine phosphatases, exaggerated mitochondrial ROS generation results in perturbation of the PI3K pathway, insulin resistance 80 and deregulation of uncoupling proteins (UCP) that normally act as oxidative stress protective agents. Subsequently mitochondrial oxidative phosphorylation is uncoupled from ATP synthesis, followed by an increase in the cellular NADH/NAD^+^ ratio through heightened glycolysis. Furthermore, adipocyte O_2_ consumption may be inhibited by ROS, since pyruvate and N‐acetyl cysteine stimulated respiration in adipocytes in vitro. ROS inhibition of O_2_ consumption may explain the difficulty in identifying effective strategies to increase fat burning in adipocytes. Stimulating fuel oxidation in adipocytes by decreasing ROS may provide a novel means to shift the balance from fuel storage to fuel burning. Aggravation of mitochondrial dysfunction in a variety of cells, combined with endoplasmic reticulum stress in the adipocyte with related unfolded protein response, converge in a systemic pro‐inflammatory state 81.

In HUVECs, apoptosis induced by high glucose was inhibited dose‐dependently by quercetin sulfate/glucuronide (300 nM), in addition to H_2_O_2_ quenching, inhibition of JNK and of caspase‐3 activity 82. H_2_O_2_‐decreased total intracellular glutathione and JNK and p38 MAPK phosphorylation were blocked by quercetin ≥10 μM 83. Quercetin reduced high glucose‐induced HUVEC proliferation, and increased apoptosis. HSP27 phosphorylation is important in mediating the proliferation and apoptosis of HUVECs induced by high glucose, and PI3K/Akt and ERK1/2 are important signalling pathways that contribute to HSP27 phosphorylation. Quercetin or quercetin‐3ʹ‐*O*‐sulfate (10 μM) inhibited, by an endothelium‐independent action, receptor‐mediated contractions of the porcine isolated coronary artery 84. (–)‐Epicatechin and (+)‐catechin (0.5–10 μM), and to a lesser extent their methylated and glucuronidated metabolites, protected against H_2_O_2_‐induced intracellular oxidative stress 85. Quercetin is rapidly taken up by cells and accumulates in various cell compartments, with remarkable concentrations found in mitochondria 86, 87. Intra‐mitochondrial quercetin appears to be functional for prevention of mitochondrial damage as well as for redistribution to the cytosol, when the fraction of the flavonoid therein retained is progressively consumed either by cell‐permeant oxidants such as peroxynitrite or by activation of plasma membrane oxidoreductases. It has also been postulated that inflammation enhances intracellular β‐glucuronidase leading cells exposed to glucuronide conjugates of quercetin to release the more bioactive aglycone. The same situation could apply for other (poly)phenols 88, 89. Extracellular β‐glucuronidase secreted from macrophages and in the vascular bed can also lead to de‐conjugation of the glucuronide conjugates of (poly)phenols and deliver the aglycone at a higher local concentration, which due to its higher lipid solubility accumulates in tissues 89, 90. In support of this notion, a recent acute human study reported effects of quercetin in hypertensive subjects related to increased plasma glutathione levels, an antioxidant biomarker; these effects were proportional to the bioavailability of quercetin and the rate of deconjugation of the glucuronide in tissues 91. Mitochondrial proton F_0_F_1_‐ATPase synthesises ATP during oxidative phosphorylation, and partial inhibition of ATPases can affect the energy balance and metabolism in cells. Quercetin has been known to interact with ATPases since the 1970s 92, 93, and resveratrol is also an inhibitor 94. After treatment with quercetin for 4 weeks at 20 mg/kg/day, kidney Na, K‐ATPase affinity for sodium was decreased in both normotensive and hypertensive rats 95. The inhibition constant (competitive) for quercetin on Ca^2+^‐ATPase from rabbit skeletal muscle was 8.7 μM 96.

### 2.4 Effects on insulin resistance through *β*‐cell function

It has been suggested that pancreatic islet inflammation is associated with very early insulin resistance, while there is strong evidence showing that the recruitment and activation of IL1β‐producing macrophages mediate islet inflammation. In *β*‐cells, mitochondrial activity is more than two times greater than in any other cell type as mitochondrial ATP generation plays a pivotal role in insulin secretion of pancreatic *β*‐cells. Increased mitochondrial ATP production in response to hyperglycaemia closes the ATP sensitive potassium channels, leading to membrane depolarization, opening of voltage‐sensitive calcium channel, calcium ion influx and insulin granule exocytosis 97. The increased expression of UCP2 is also responsible for affecting glucose‐induced insulin secretion due to the reduction of ATP production 81. *β*‐cells are poor in antioxidant enzymes such as superoxide dismutase, glutathione peroxidase and catalase, and therefore particularly sensitive to ROS and reactive nitrogen species 98 amplified by hyperglycaemia and increased levels of fatty acids. Apoptosis of *β*‐cells further accentuates the vicious cycle linking *β*‐cell failure, mitochondrial dysfunction and insulin resistance through pronounced oxidative damage. To counteract these insults, most cells, including *β*‐cells, have intricate mechanisms of defence against ROS toxicity and among these, the transcription factor NF‐E2–related factor 2 (Nrf2) is a pivotal component. In response to oxidative stress, activation of Nrf2 dramatically increases intracellular antioxidant potential by directly increasing the transcription of many so‐called antioxidant enzymes.

(Poly)phenols and pancreatic β‐cell function have been recently reviewed 99. Green tea catechins were reported to have a beneficial role in regulation of insulin secretion in *β* cells isolated from fed adult male Wistar rats. EGCG and epicatechin gallate (but not epigallocatechin or epicatechin, source not specified) were potent inhibitors of glutamate dehydrogenase (GDH) activity with ED_50_ values of ∼300 nM. Glutamate serves as a mitochondrial intracellular messenger when glucose is being oxidized, and EGCG did not affect glucose‐stimulated insulin secretion under high energy conditions where GDH was fully inhibited 100. Cai et al. 101 evaluated the effect of EGCG on glucose‐induced toxicity in a rat pancreatic β‐cell line, rat insulinoma (RIN)‐m5F cells, and showed that EGCG (0.1 and 10 μM) treatment improved insulin secretory function and viability of β‐cells under conditions of glucotoxicity. These effects were at least partly mediated through increased expression of IRS‐2, Akt and FOXO1 and an enhancement of mitochondrial mass and functional integrity in high glucose.

Apart from enhancing mitochondrial status, other protective effects of (poly)phenols such as flavanols, quercetin, luteolin and others in vitro have been recently reviewed 102 and seem to be mainly mediated through suppression of inflammatory cytokine production and ROS/reactive nitrogen species. Direct binding of (poly)phenols to receptors involved in signalling pathways discussed earlier and enzyme inhibition of oxidative enzymes account for some of the mechanisms involved. However, there are few human studies assessing β‐cell functionality, as relevant biomarkers have not been strictly defined. Recently curcumin has emerged as an attractive nutritional bioactive in the field of diabetic nutrition. This assertion comes after a 9‐month study, involving a pre‐diabetic population, which demonstrated that curcumin treatment could not only lower haemoglobin A1c (HbA1c) and homeostasis model assessment of insulin resistance (HOMA‐IR) levels (a measure of insulin sensitivity), but also decelerate the deterioration of pre‐diabetes to type 2 diabetes 103. Curcumin (1–100  pM) and resveratrol (0.1–10  μM) were reported to enhance pancreatic *β*‐cell function by regulating the activity of phosphodiesterases, which degrade cAMP and cGMP, thereby modulating various cellular signalling pathways previously linked to regulation of insulin secretion in islets 104. Linking these data to the human study outcomes remains quite challenging as curcumin is quite unstable under certain cell culture conditions, and the controversy surrounding resveratrol clinical outcomes warrants more well‐controlled mechanistic work. Frequent spikes of post‐prandial glucose can affect insulin secretion and *β*‐cell dysfunction accelerating the onset of diabetes. Towards that direction, nutritional interventions with low glycaemic index foods could lead both to enhancing insulin secretion and suppressing oxidative species in the vasculature, through a delay in the release of glucose to the bloodstream and available substrate for oxidation in mitochondria, at the same time of a high lipid bolus 19.

## 3 Effects of (poly)phenols on blood pressure, platelet aggregation and endothelial dysfunction via interactions of (poly)phenols with endothelial and smooth muscle cells

It remains unclear whether elevated levels of free radicals initiate the development of hypertension or are a consequence of the disease process itself, or both 105. Vascular alterations may be potentiated in several ways, including direct damage to endothelial and vascular smooth muscle cells (VSMC), as the endothelium plays a crucial role in modulating vascular tone by synthesizing and releasing endothelium‐derived relaxing factors, including vasodilator prostaglandins, NO and endothelium‐dependent hyperpolarization (EDH) factors, as well as endothelium‐derived contracting factors. For example, endothelium‐derived NO inhibits growth factor‐stimulated proliferation and migration of VSMC 106. As early as in 1988 107, 108, it was postulated that a diffusible substance released by the endothelium causes hyperpolarization and relaxation of the underlying VSMC, attributed to the existence of putative EDH factors. It is widely accepted that the nature of EDH factors varies depending on species and vascular beds examined, including epoxyeicosatrienoic acids, metabolites of arachidonic P450 epoxygenase pathway, electrical communication through gap junctions, K^+^ ions, hydrogen sulfide (H_2_S), and H_2_O_2_ (reviewed in 109).

NO predominantly modulates the tone of large conduit vessels and the contribution of NO decreases as the vessel size decreases, whereas that of EDH increases as the vessel size decreases, a phenomenon that is well preserved from rodents to humans. Thus, EDH, rather than NO, plays a dominant role in small resistance vessels where blood pressure and organ perfusion are finely regulated. It has been demonstrated that Akt1 is involved in the depolarisation process preceding the activation of NADPH oxidase (NOX) and partnering with PKC to mediate these effects 97.

Several recent reviews have summarised the effect of (poly)phenols and (poly)phenol‐rich foods on blood pressure, platelet aggregation and endothelial dysfunction in volunteers 19, 110, 111, 112, 113, 114, and the evidence has resulted in one of the few current European Food Safety Authority (EFSA) allowed claims on (poly)phenols ((‐)‐epicatechin). In a randomized, placebo‐controlled, crossover trial on healthy men, 200 mg quercetin increased plasma *S*‐nitrothiols, plasma nitrite and urinary nitrate, and reduced both plasma and urine endothelin‐1, but did not change isoprostanes 115. In the same study, (–)‐epicatechin lowered plasma endothelin‐1, *S*‐nitrothiols and nitrite, and urinary nitrate, but EGCG at the same dose did not show any of these changes 115.

**Figure 1:**
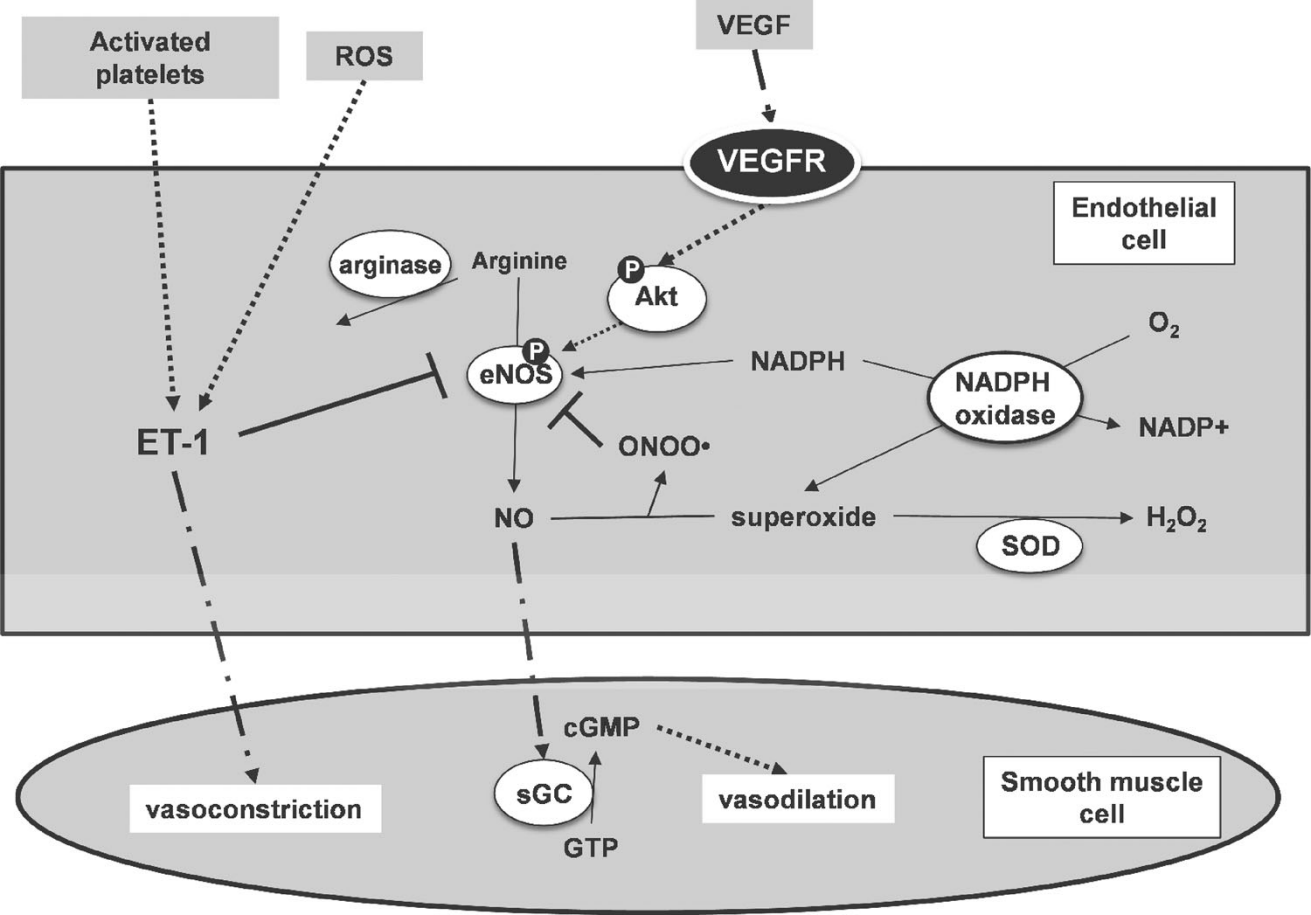
Selected metabolic pathways involved in vasoconstriction and vasodilation where there is evidence for the action of (poly)phenols. sGC, soluble guanylyl cyclase; VEGFR, vascular endothelial growth factor receptor; VEGF, vascular endothelial growth factor; SOD, superoxide dismutase; eNOS, endothelial nitric oxide synthase. Thin arrows show direct reactions, dotted arrows indicate pathway with multiple reactions/interactions, and a dot‐dash line indicates movement of molecules.

Many animal studies have also been performed, for example (–)‐epicatechin (6 mg/kg body weight) decreased systolic blood pressure in spontaneously hypertensive rats, but there was no effect in normotensive Wistar–Kyoto rats 116. However, the mechanism of action is complex, and is likely to result from small effects on multiple targets, since many factors regulate these processes. Realistic potential targets affected by (poly)phenols are NOX, endothelin production, arginase, eNOS and NO metabolism (Fig. 1) and these are discussed below. In healthy volunteers, a high flavanol cocoa drink led to a lower arginase activity in erythrocytes 117. Many of the experiments on endothelial function have been performed on HUVECs in vitro, and some are on the underlying smooth muscle cells. Signals between the two cell types are important for determining vasoconstriction, associated with increased blood pressure, and vasodilation, and resulting lowered blood pressure. Some of the molecular mechanisms, focusing on those that might be modulated by (poly)phenols, are shown in Fig. 1. In addition, HUVECs exhibit some capacity to metabolise (poly)phenols. For example, (–)‐epicatechin is methylated and metabolised to 3‐methyl‐(–)‐epicatechin‐7‐*O*‐glucuronide and 3‐methyl‐(–)‐epicatechin‐7‐*O*‐sulfate 118. This metabolism can modify the biological activity of the parent (–)‐epicatechin 119.

### 3.1 eNOS and NO bioavailability

Although the underlying cause of endothelial dysfunction is multifactorial, a key player is impairment of NO bioavailability. NO is considered a vasodilator molecule, produced by the conversion of L‐arginine into L‐citrulline via a family of NO synthases (NOSs). There are three NOS isoforms, including neural NOS (nNOS, NOS1), inducible NOS (iNOS, NOS2) and endothelial NOS (eNOS, NOS3, Ca^2+^‐calmodulin‐dependent isoform). The latter is the dominant NOS isoform in blood vessels 120 and is the most important in generating H_2_O_2_/EDH in the endothelium as mentioned above. NO and eNOS also participate in the neo‐vascularization process, via mobilization of progenitor cells from brown marrow stem cell niches to the peripheral circulation, where they are involved in vascular repair at sites of injury through their differentiation in mature endothelium. The diverse roles of NOS systems in the endothelium depend on blood vessel size; NOS mainly serves as an NO‐generating system to elicit soluble guanylate cyclase (sGC)‐cyclic guanosine monophosphate (cGMP)‐mediated relaxations in large conduit vessels, and a superoxide‐generating system to cause EDH/H_2_O_2_‐mediated responses in small resistance vessels 121. The potential importance of the physiological balance between NO and EDH/H_2_O_2_ through the diverse functions of the endothelial NOS system is reflected in the commonly reported failure to show effects in clinical trials with antioxidant approaches.

(Poly)phenols have been reported to have several effects on these systems. Quercetin aglycone (but not conjugates) significantly reduced both eNOS protein and gene expression in HUVEC, as did TNFα. In the presence of TNFα, the aglycone caused further reductions in eNOS, whereas the metabolites were without effect in either TNFα ‐stimulated or unstimulated cells 122. HUVECs were incubated with platelets, pre‐treated with various doses of (poly)phenols, from peripheral artery disease patients. This gave a decrease of sCAMs release and an increase of p‐eNOS and NO bioavailability reducing endothelial activation induced by activated platelets 123. Hesperetin increased NO release from HUVECs in a dose‐dependent manner and up‐regulated eNOS expression 124. Hesperetin stimulated phosphorylation of Src, Akt, AMPK and endothelial NO synthase to increase NO production. Hesperetin attenuated the TNFα‐increased adhesion of monocytes and expression of vascular cell adhesion molecule‐1 26. Effects of quercetin on eNOS are contradictory, but appear to be concentration dependent. At lower concentration (<10 μM), eNOS activity is increased. For example, quercetin and quercetin‐3‐*O*‐glucuronide (5–10 μM) protected isolated C57BL mouse aortic rings against hypochlorous acid‐induced endothelial dysfunction, and this effect was blocked by AMPK inhibition. These compounds also activated AMPK and eNOS in human aortic endothelial cells, and increased nitrite and S‐nitrothiols in the medium 125. At higher concentrations of quercetin (30–100 μM), eNOS activity was decreased in bovine aortic endothelial cells by decreasing Ser617 phosphorylation, and also ser473 Akt phosphorylation 126. Increasing eNOS results in an increase in NO. For example, in endothelial cells, quercetin induced phosphorylation of eNOS (ser1179), PKA, Akt and ERK1/2, and increased NO concentration 127. In vivo, in spontaneously hypertensive rats, quercetin reduced the increase in blood pressure and heart rate, and prevented the increase in NADPH‐induced superoxide production and lowering of eNOS activity (although eNOS protein was increased in this model in contrast to the activity) 128.

### 3.2 Arginase

In several cell types, L‐arginine transport has been considered as a rate limiting factor for NO synthesis. HUVECs, endothelial cells from human placental microvessels, human saphenous veins and human endothelial progenitor cells (hEPC) are all able to transport L‐arginine and synthesize NO 129. Emerging evidence suggests that upregulation of arginase is of central importance for reduced NO bioavailability due to competition for the substrate L‐arginine between arginase and the eNOS. Arginase is also associated with increased oxidative stress, further impairing NO bioavailability. Upregulation of arginase has thereby been suggested to be a key factor driving endothelial dysfunction in diabetes. There are only limited examples where (poly)phenols affect arginase activity. In HUVECs, cocoa flavanols lowered both arginase‐2 mRNA expression and activity, and this was supported in the same paper by a study on rats, where flavanol‐rich cocoa decreased arginase activity in rat kidney 117.

### 3.3 NADPH oxidase

NADPH oxidase (NOX) is a major source of ROS in the vasculature, especially in response to high glucose. NOX1 was shown to be involved in angiotensin‐II‐mediated hypertension using NOX1‐deficient mice 130. Sustained activation of NOX in diabetes leads to lowered intracellular NADPH, an essential cofactor for eNOS 131. High glucose in HUVECs leads to upregulation of the p47^phox^ subunit of NOX through NF‐κB activation 132, 133 and oxidative stress markers. (–)‐Epicatechin glucuronide and 3ʹ/4ʹ‐methyl (–)‐epicatechin, but not (–)‐epicatechin, inhibited NOX activity in HUVEC cells 119. Quercetin and quercetin‐3‐*O*‐glucuronide (1 μM) inhibited NOX activity in vascular smooth muscle cells ex vivo from both spontaneously hypertensive rats and normotensive Wistar Kyoto rats, and this effect was abolished by the β‐glucuronidase inhibitor, saccharolactone, indicating that the active aglycone is generated in situ 134. (–)‐epicatechin (0.3–10 μM) increased NO in HUVECs but did not affect eNOS mRNA 135. HUVECs treated with oxLDL, but not with LDL, exhibited decreased intracellular NO and superoxide overproduction, prevented by quercetin or resveratrol. Oxidised LDL also up‐regulated leukocyte recruitment and adhesion genes, which was abolished by resveratrol, but not quercetin (10 μM), which even increased some of the genes 136. *O*‐methylated (–)‐epicatechin suppressed superoxide generation through inhibition of endothelial NOX activity, and the effect was even more potent than the commonly used positive control, apocynin 137.

### 3.4 Endothelin

Endothelin‐1 (ET‐1) is a small peptide with potent vasoconstrictor activity, and its production by endothelial cells is stimulated by several factors including insulin, hypoxia, oxidized LDL, or pro‐inflammatory cytokines 138. ET‐1 also modulates cell growth, and is possibly involved in the development and progression of atherosclerosis and hypertension. In HUVECs, resveratrol and quercetin (0.1 μM), but not ferulic acid, decreased expression of endothelin‐1 139. Quercetin and quercetin‐3ʹ‐*O*‐sulfate (1 μM) attenuated ET‐1‐induced endothelial dysfunction in Wistar rat thoracic aortic rings, and at higher concentrations, together with quercetin‐3‐*O*‐glucuronide, inhibited NOX‐derived superoxide release 140.

Thrombin and activated platelets stimulated the release of ET‐1 from HUVECs, and this was inhibited by quercetin (0.5–50 μM) that also promoted the formation of cGMP 141.

Quercetin aglycone (but not conjugates) significantly reduced ET‐1 expression in TNFα stimulated and unstimulated HUVECs, but quercetin‐3′‐*O*‐sulfate caused a moderate increase of ET‐1 in TNFα stimulated cells 122. ET‐1 increased the phenylephrine‐induced contractile response in the aortic rings from male Wistar rats, and this was attenuated by quercetin and isorhamnetin (1 and 10 μM). In the same system, ET‐1 induced upregulation of NOX p47phox protein and intracellular superoxide and eNOS uncoupling, again attenuated by quercetin 142.

### 3.5 ACE inhibition

Endothelial cells express angiotensin‐converting enzyme (ACE) and angiotensin AT1 and AT2 receptors. ACE activity inhibition may result in a decrease of blood pressure by causing blood vessel constriction through conversion of angiotensin I to angiotensin II, which binds to AT1 receptors and receptors expressed on smooth muscle cells, and also stimulates the release of aldosterone 142. Although angiotensin II can also induce vasorelaxation by stimulating AT2 receptors and upregulating eNOS and inducible NO synthase (iNOS) expression, vasoconstriction is predominant in most circumstances as it reduces eNOS‐derived NO by promoting eNOS uncoupling through monocyte‐dependent S‐glutathionylation 143. Furthermore, angiotensin II and aldosterone promote inflammation by inducing COX‐2 expression, fibrosis and endothelial dysfunction with upregulation of adhesion molecules via TNFα, considered important for the development of atherosclerotic lesions and plaque rupture 144.

There are limited convincing reports of inhibition of ACE activity by (poly)phenols at physiologically relevant concentrations. ACE was not inhibited by (supra)physiological concentrations of flavanols and procyanidins 145, 146. Quercetin‐3‐*O*‐glucuronide (1 and 10 μM) blocked angiotensin‐II induced JNK activation in rat aortic smooth muscle cells 147. Similarly, the reduction in the angiotensin II‐induced endothelial expression of chemokines and adhesion molecules by EGCG occurs via ERK1/2 and p38 inhibition 148. A flavonoid‐rich apple peel extract, and also quercetin‐3‐*O*‐glucuronide and quercetin‐3ʹ‐*O*‐sulfate (indicated as the 3‐*O*‐sulfate in the paper but most probably is the 3ʹ‐*O*‐sulfate), inhibited ACE, but a high concentration was needed (IC_50_ values of 27 and 131 μM, respectively) 149.

### 3.6 Protease activity

The extracellular matrix (ECM) plays an important structural and functional role in maintaining proper contractility and integrity of arterial walls. The composition and structure of ECM undergoes significant changes during the atherosclerotic process as a result of compromised regulation of the matrix metalloproteinase (MMPs) network and modifications of matrix component synthesis and deposition. Denudation of the arterial wall surface from endothelial cells and exposure of the underlying ECM to blood dramatically increases monocyte binding to injured sites and their arterial wall invasion. Leukocyte adhesion to ECM components is mediated by cell surface–expressed integrins with different specificity and affinity 47. Angiotensin II increases receptors for vascular endothelial growth factors and MMPs that may account for increases in endothelial permeability and vascular remodelling 150. Genistein, apigenin and 3‐hydroxyflavone inhibited in vitro angiogenesis, in part via preventing vascular endothelial growth factor/basic fibroblast growth factor (VEGF/bFGF)‐induced MMP‐1 and urokinase‐type plasminogen activator (uPA)‐plasmin system expression and the activation of pro‐MMP‐2, and via modulating their inhibitors, TIMP‐1 and ‐2, and PAI‐1 151. (+)‐Catechin, (–)‐epicatechin, B2 dimer and gallic acid (<1 μM) inhibited dipeptidyl‐peptidase 4 (DPP4) activity in HUVEC by 5–10 % 152.

Angiotensin II–induced contractile activity of cultured human ASMC embedded in a collagen matrix was inhibited by catechins, dependent on the number of gallate groups, and this correlated with inhibition of matrix metalloproteinase‐2 expression 153.

### 3.7 Telomere length, oxidative stress and sirtuins; role in vascular ageing

The emerging role of cellular DNA damage and the link to endothelial vascular complications stems from association studies showing that telomere length reflects the lifelong accumulating burden of increased oxidative stress and inflammation 154.

The epigenetic changes of histone and nonhistone protein deacetylation are catalysed by sirtuins (SIRTs), a family of proteins with seven isoforms that all possess a common, highly conserved catalytic domain and an NAD^+^‐binding site. The role of SIRTs in vascular homeostasis has been recently reviewed 106, 155 and especially in relation to the actions of the (poly)phenol resveratrol; a known SIRT1 activator in various models and settings 156, 157. SIRT1 is highly expressed in the vasculature during blood vessel growth and partly controls the angiogenic activity of endothelial cells via a deacetylation‐dependent inactivation of FoxO1, a crucial negative transcriptional regulator of blood vessel development; thus SIRT1 promotes cell survival under oxidative stress and inhibits apoptosis. Inhibition of SIRT1 induces premature senescence‐like growth arrest in HUVECs whereas overexpression or activation of SIRT1 prevents premature senescence induced by H_2_O_2_. In addition, protection against oxidative stress‐induced cellular senescence is mediated by downregulation of p66Shc, a stimulator of mitochondrial production of ROS and against oxidative stress‐induced EPC apoptosis by promoting the ubiquitination and degradation of FoxO3a.

SIRT1 has also been linked to vascular health by activation of eNOS through deacetylation of lysine residues in the calmodulin‐binding domain. In SIRT1, SMC acts as a modulator of neointima formation and protects against DNA damage, medial degeneration, atherosclerosis and hypertension via repression of AP‐1 activity and decreased expression of cyclin D1 and MMP‐9 158. In endothelial cells, SIRT1 is reported to act as an NF‐κB suppressor and its downstream pro‐inflammatory signalling pathways leading to the reduced expression of chemokines such as TNFα, MCP‐1 and interleukins. SIRT1 activation was shown to inhibit cholesterol uptake and macrophage foam cell formation impeding infiltration of inflammatory cells to the sub‐endothelial space. The beneficial effects of ACE inhibitors in endothelial cells were also partly ascribed to a p38‐SIRT1 crosstalk 142. However it is worth noting that, under basal conditions, SIRT1 may undergo post‐translational modifications that can restrict its activities, such as serine phosphorylation on various sites (ser27, ser47, ser659 and ser661) that is mainly regulated by protein kinase CK2 and JNK 159.

The “French paradox” was inaugurated in the early 1990s 160 mainly related to the conundrum of a diet high in atherogenic factors with a moderate consumption of alcohol. Wine (poly)phenols have attracted substantial research interest since, in 2003, Howitz et al. 161 reported resveratrol as a potent activator of SIRT1; and although the data has been challenged 162, extensive in vitro and animal research since suggests a role for (poly)phenols in inflammation through interplay with SIRT1 and associated pathways 156, 159 or through indirect enhancement of oxidative status in compromised models of disease. In a recent study in endothelial cells following palmitate‐induced insulin resistance, 0.1–10 μM resveratrol restored the IRS‐1/Akt/endothelial NO synthase signalling pathway by suppressing IKKβ/Nf‐κB phosphorylation, as well as TNFα and IL‐6 production. Furthermore, resveratrol effectively inhibited the mitogenic actions of insulin by decreasing the secretion of ET‐1 and PAI‐1, and positively regulated SIRT1 activation, which contributed to the inhibition of inflammation implicated in endothelial insulin resistance. Stimulation with palmitate and long‐term‐fructose feeding introduced impaired insulin‐mediated vessel dilation in rat aorta, whereas pre‐treatment of excised and prepared aortic rings with resveratrol (0.1–10 μM) or treatment of rats with 5 or 20 mg/kg resveratrol produced similar effects 163.

Other studies also showed that 2.5 mg/kg body wt/day of resveratrol for 15 days ameliorated endothelial dysfunction by regulating VEGF, eNOS, caveolin‐1 and haem oxygenase (HO)‐1 164 in diabetic rats, while a dose of 20 mg/kg resveratrol for two weeks led to neovascularization of the hyper‐cholesterolaemic myocardium of rats and conferred protection against myocardial injury caused by ischemia‐reperfusion through the same mechanism 165. These studies are only a few of many other animal studies and others in vitro in the literature showing beneficial effects of resveratrol in the endothelium through NO‐related mechanisms 166, 167.

Nevertheless, data from human studies linking to positive translational effects remain scarce. A study recently showed no beneficial effects on anthropometric measurements, insulin resistance markers, lipid profile and blood pressure following a 12‐week supplementation of 500 mg resveratrol in a randomised double‐blinded placebo‐controlled clinical trial of 50 NAFLD patients 168. In contrast, 6 weeks supplementation with 500 mg resveratrol improved quality of life and clinical manifestation of colitis at least partially through inflammation reduction in patients with ulcerative colitis 169. Resveratrol supplementation significantly decreased serum inflammatory markers, activity of NF‐κB in peripheral blood mononuclear cells (PBMC) and plasma levels of TNFα and hs‐CRP. In another randomized, double‐blind, placebo‐controlled trial of 12 weeks of resveratrol supplementation (75 mg/day) in 30 non‐obese, postmenopausal women with normal glucose tolerance, no metabolic effects or inflammatory markers were demonstrated and resveratrol did not affect SIRT1 and other related pathways in either skeletal muscle or adipose tissue biopsies of volunteers 170. These conflicting data reflect challenges when translating pre‐clinical findings to improved patient outcomes and only modest reductions in the degree of inflammatory parameters based on direct measurement of the plasma levels of well‐known pro‐inflammatory markers have been reported 171. Other (poly)phenols such as quercetin are known to activate SIRT1in the endothelium to a lesser extent and the effects are dose‐dependent. For example, quercetin (2.5–10 μM) suppressed oxLDL‐induced endothelial oxidative injuries by activating SIRT1 and modulating the AMPK/NADPH oxidase/AKT/endothelial NO synthase signalling pathway 172. However these results have not been tested in the clinical setting. Other indirect mechanisms through which quercetin and other (poly)phenols such as luteolin and apigenin could contribute to enhancing NAD^+^ availability and activating SIRT1 relieving mitochondrial stress is through inhibition of NAD^+^ consuming enzymes such as the poly(ADP‐ribose) PAR polymerase‐1 CD38 NAD^+^ase 173, 174, 175.

## 4 Modulation of intracellular superoxide and plasma uric acid via inhibition of xanthine oxidoreductase

**Figure 2:**
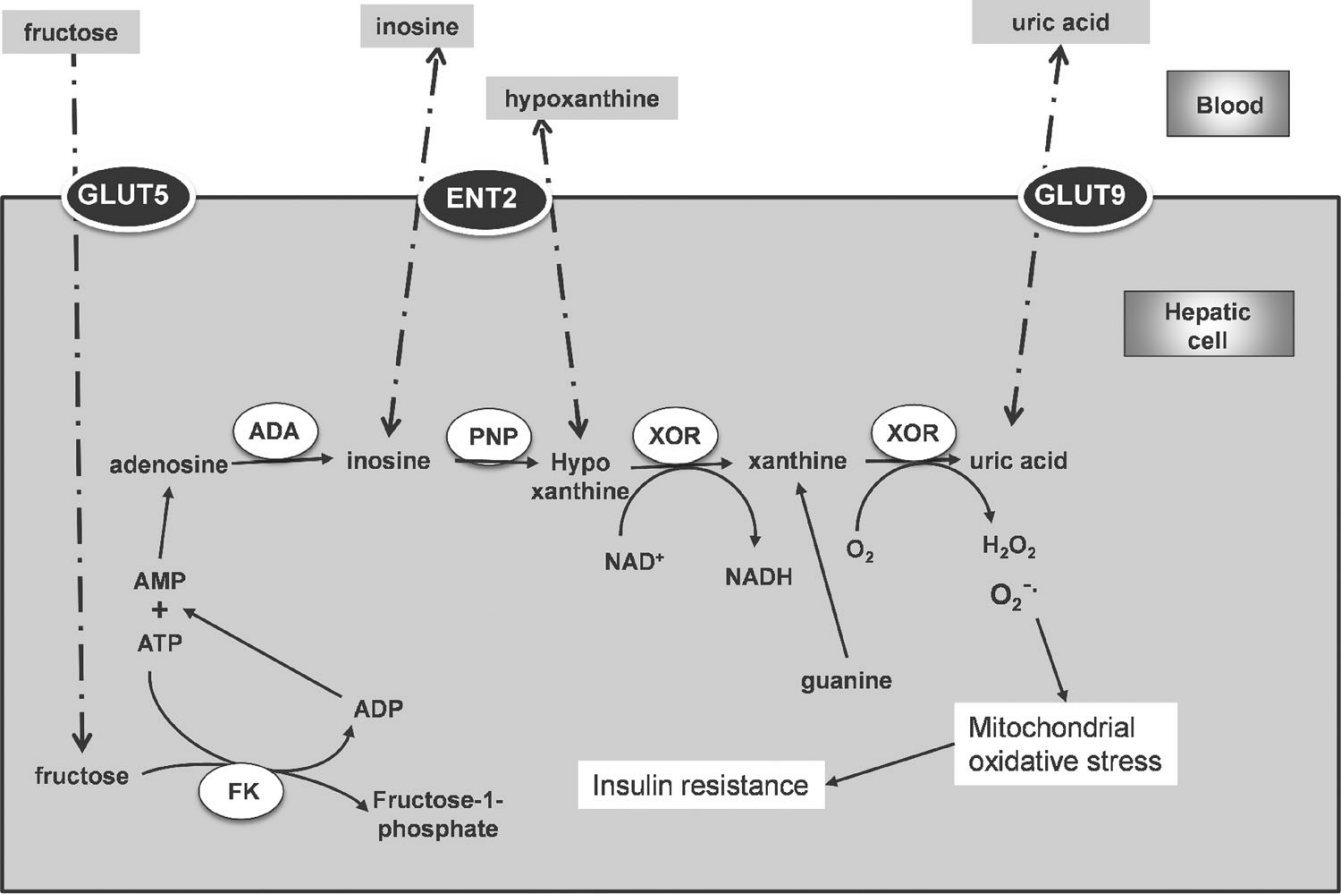
Selected metabolic pathways leading to generation of uric acid in the liver. ADA, adenosine deaminase; PNP, purine nucleoside phosphorylase; FK, fructokinase; ENT2, SLC29A2; GLUT5, SLC2A5; GLUT9, SLC2A9. Thin arrows show direct reactions, dotted arrows indicate pathway with multiple reactions/interactions, and a dot‐dash line indicates movement of molecules.

Elevated blood uric acid (hyperuricaemia) is a major risk factor for gout 160, diabetes 176, obesity 177 and hyperglycaemia 178, 179, and is exacerbated by dietary purines, alcohol and fructose 180, 181, 182. Insulin resistance is also related to hyperuricaemia 183 and metabolic conditions such as non‐alcoholic fatty liver disease increase plasma uric acid 184. Uric acid is a product of several endogenous cellular biochemical reactions (Fig. 2), where xanthine oxidoreductase (XOR; dehydrogenase: EC 1.1.1.204; oxidase: EC 1.1.3.22) is the final step in the formation of uric acid from xanthine and hypoxanthine, and is a source of intracellular superoxide. It is a complex protein and requires molybdenum, flavin adenine dinucleotide and iron‐sulfur redox complexes for activity, and is particularly abundant in liver and small intestine 185. Whether the product is hydrogen peroxide or superoxide depends on the biochemical form of the enzyme generated through intracellular processing. The product, superoxide, reacts with NO to form peroxynitrite, which has several deleterious activities, and also changes the bioavailability of NO. In endothelial cells, peroxynitrite also oxidises tetrahydrobiopterin (a cofactor for eNOS), causing uncoupling of eNOS and endothelial dysfunction 131. Over‐activity of xanthine oxidoreductase leads to production of excess uric acid and superoxide, both with negative implications.

Quercetin and other flavonoids were identified as inhibitors of XOR over 60 years ago 186, and subsequently isorhamnetin and quercetin glucuronides were also shown to inhibit potently 187, 188. Interaction with XOR is strongly dependent on the chemical structure and shape of the molecule, since chrysin, luteolin, kaempferol, quercetin, myricetin and isorhamnetin, were potent inhibitors, whereas isoflavones and anthocyanidins were less effective 189. For apigenin, the bicyclic benzopyranone ring stacked with the Phe^914^ residue of XOR, the phenolic group interacted with multiple XOR hydrophobic amino acids, and the 3‐hydroxyl group as present in quercetin slightly diminished the binding strength 190. The important chemical aspects of flavonoids required for XOR inhibition have recently been summarised 191.

Serum uric acid in mice given a very high dose of quercetin (100–400 mg/kg body weight) were elevated, but these authors failed to show inhibition of XOR by quercetin in vitro, despite observing allopurinol inhibition as a positive control 192. On the other hand, in rodents made hyperuricaemic by intraperitoneal injection of potassium oxonate, quercetin (5 mg/kg) for 2 weeks significantly reduced serum uric acid, paralleled by an inhibition of hepatic XOR activity 193. Oral administration of various flavonoids including quercetin (50 and 100 mg/kg) for 3 days to potassium oxonate‐induced hyperuricaemic mice reduced both blood and hepatic uric acid, again with inhibition of XOR 194.

Very few human intervention studies have been reported on (poly)phenols or (poly)phenol‐rich foods where plasma uric acid is the primary outcome. Our recent study on mildly hyperuricaemic males showed an 8% reduction in plasma uric acid after consumption of 500 mg quercetin aglycone per day for 1 month 195, with the equivalent bioavailability of ∼100 g red onions 196. In studies where uric acid was measured, but not as a primary outcome, the results are mixed, and none of the studies selected volunteers with higher than average plasma uric acid. In healthy volunteers, supplementation of diets with 50, 100 or 150 mg/day quercetin aglycone for 2 weeks 197, or with a blueberry–apple juice mixture (97 mg quercetin/litre) for 4 weeks 198 gave no change in plasma uric acid. In stable type 2 diabetic patients, 76–110 mg of flavonols (mostly quercetin from 400 g of onions, and tomato sauce) and six cups of tea daily for two weeks gave no change in plasma uric acid 199. Likewise, in smokers, six cups (900 mL) of black or green tea, or a supplement of 3.6 g of green tea (poly)phenols per day for 4 weeks elicited no change in plasma uric acid 200.

Although the number of relevant studies is too small to conclude, it is tempting to speculate that the effect of (poly)phenols, in particular quercetin, is to lower the blood levels of uric acid only when elevated levels are present at the start of the intervention. This hypothesis needs to be thoroughly tested, and we suggest that this is a priority for future work.

## 5 Future perspectives

Once early vascular ageing is defined, it remains to be determined whether interventions, such as by targeting the different pathways or elements, could slow down the process and prevent further deterioration. Pharmacological agents, including angiotensin‐converting enzyme inhibitors, angiotensin II type 1 blockers, aldosterone antagonists and statins, are able to reduce arterial stiffness. However there are still no pharmaceutical agents that specifically target the vascular endothelium, and the main clinical advice remains to increase physical activity in combination with nutritional interventions. Although in vivo detection and targeting of senescent endothelial cells are still being investigated, it is likely that therapeutic strategies based on enhancing endothelial stress and preventing oxidative damage through the molecular avenues discussed would constitute an innovative treatment, perhaps by nutritional means, for slowing down vascular ageing. After all, the evidence that (poly)phenols may deliver a maximised effect at sites of inflammation has been proposed for quercetin as discussed above. In addition, nutritional intake implies that the body is exposed to (poly)phenols on a regular basis, which can exert an effect without any need for a “treatment” as such. One of the key aspects is that effects are most predominant when one or more biomarkers are suboptimal. Nevertheless, future studies should have enough power to detect differences, a range of doses and combinations should be tried, long‐term studies should be designed and the participants should be well characterized. These studies should then be backed up by mechanistic studies in vitro.
